# Evaluation of the Anti-Inflammatory, Antioxidant and Immunomodulatory Effects of the Organic Extract of the Red Sea Marine Sponge *Xestospongia testudinaria* against Carrageenan Induced Rat Paw Inflammation

**DOI:** 10.1371/journal.pone.0138917

**Published:** 2015-09-30

**Authors:** Nagla A. El-Shitany, Lamiaa A. Shaala, Aymn T. Abbas, Umama A. Abdel-dayem, Esam I. Azhar, Soad S. Ali, Rob W. M. van Soest, Diaa T. A. Youssef

**Affiliations:** 1 Department of Pharmacology and Toxicology, Faculty of Pharmacy, King Abdulaziz University, Jeddah, Saudi Arabia; 2 Department of Pharmacology and Toxicology, Faculty of Pharmacy, Tanta University, Tanta, Egypt; 3 Natural Products Unit, King Fahd Medical Research Center, King Abdulaziz University, Jedda, Kingdom of Saudi Arabia; 4 Special Infectious Agents Unit, King Fahd Medical Research Center, King Abdulaziz University, Jeddah, Kingdom of Saudi Arabia; 5 Biotechnology Research Laboratories, Gastroenterology Surgery Center, Mansoura University, Mansoura, Egypt; 6 Animal Facility Unit, King Fahd Medical Research Center, King Abdulaziz University, Jeddah, Saudi Arabia; 7 Medical Laboratory Technology Department, Faculty of Applied Medical Sciences, King Abdulaziz University, Jeddah, Saudi Arabia; 8 Anatomy Department (Cytology and Histology), Faculty of Medicine, King Abdulaziz University, Jeddah, Kingdom of Saudi Arabia; 9 Naturalis Biodiversity Center, Deptartment of Marine Zoology, P.O. Box 9517, 2300 RA Leiden, The Netherlands; 10 Department of Natural Products, Faculty of Pharmacy, King Abdulaziz University, Jeddah, Kingdom of Saudi Arabia; Shanghai University of Traditional Chinese Medicine, CHINA

## Abstract

Marine sponges are found to be a rich source of bioactive compounds which show a wide range of biological activities including antiviral, antibacterial, and anti-inflammatory activities. This study aimed to investigate the possible anti-inflammatory, antioxidant and immunomodulator effects of the methanolic extract of the Red Sea marine sponge *Xestospongia testudinaria*. The chemical composition of the *Xestospongia testudinaria* methanolic extract was determined using Gas chromatography-mass spectroscopy (GC-MS) analysis. DPPH (2, 2-diphenyl-1-picryl-hydrazyl) was measured to assess the antioxidant activity of the sponge extract. Carrageenan-induced rat hind paw edema was adopted in this study. Six groups of rats were used: group1: Control, group 2: Carrageenan, group 3: indomethacin (10 mg/kg), group 4–6: *Xestospongia testudinaria* methanolic extract (25, 50, and 100 mg/kg). Evaluation of the anti-inflammatory activity was performed by both calculating the percentage increase in paw weight and hisopathologically. Assessment of the antioxidant and immunomodulatory activity was performed. GC-MS analysis revealed that there were 41 different compounds present in the methanolic extract. Sponge extract exhibited antioxidant activity against DPPH free radicals. *Xestospongia testudinaria* methanolic extract (100 mg/kg) significantly decreased % increase in paw weight measured at 1, 2, 3 and 4 h after carrageenan injection. Histopathologically, the extract caused a marked decrease in the capillary congestion and inflammatory cells infiltrate. The extract decreased paw malondialdehyde (MDA) and nitric oxide (NO) and increased the reduced glutathione (GSH), glutathione peroxidase (GPx), and catalase (CAT) activity. It also decreased the inflammatory cytokines, tumor necrosis factor-α (TNF-α), interleukin-1 β(IL-1β) and IL-6. The results of this study demonstrated the anti-inflammatory, antioxidant, and immunomodulatory effects of the methanolic extract of the Red Sea sponge *Xestospongia testudinaria* (100 mg/kg).

## Introduction

Inflammatory reaction is a common physiologic response which protects the host from many harmful stimuli such as toxins, local injuries and pathogens [1]. Uncontrolled inflammation is considered as one of the pathophysiologic causes of most chronic diseases, including chronic inflammatory diseases, diabetes, cancer and cardiovascular diseases [2–3]. Over the years, reactive nitrogen species and reactive oxygen species (ROS) are found to play a key role in inflammation [4]. In addition, extensive scientific reports described the role played by various proinflammatory cytokines, including tumor necrosis factor-α (TNF-α), interleukin-1 β(IL-1β) and IL-6 in inflammation [5]. Nowadays, non-steroidal anti-inflammatory drugs (NSAIDs) are the most commonly prescribed therapeutics for the treatment of many inflammatory diseases [6]. However, the long-term administration of these NSAIDs causes many harmful effects including gastrointestinal ulcers and bleeding and renal damage [7]. Therefore, the need for new anti-inflammatory drugs with fewer adverse effects is mandatory [8].

Since ancient times, marine environment has been documented to be a valuable source of bioactive metabolites including antioxidants, polyunsaturated fatty acids (PUFAs) and sterols [2]. These naturally occurring bioactive substances often have no matches on earth. Sponges are found to be a rich source of bioactive compounds with antiviral, antibacterial, and anti-inflammatory activities [2,3]. The most reported anti-inflammatory mechanisms of marine sponges metabolites were, inhibition of phospholipase A2 (PLA2) (a key enzyme in the arachidonic acid cascade); and inhibition of interleukine (IL)-1 mediated prostaglandin (PG)-E2 synthesis [9]. Clathriol B was a novel polyoxygenated steroids isolated from the marine sponge Clathria lissosclera, inhibited the production of superoxide from human peripheral blood neutrophils, which is known to be implicated in the pathogenesis of inflammatory disorders [10].

Sponges of the genus *Xestospongia* (class Desmospongia, order Haplosclerida, family Petrosiidae) are a large group among coral reef communities, known as barrel sponges, located all over the Caribbean Sea and Indo-Pacific Ocean at depths greater than 10 meters [11]. Members of the genus *Xestospongia* are considered a rich source of pharmacologically active compounds [10]. Recently, sterols have been isolated from the marine sponge *Xestospongia testudinaria* [11]. In addition, many recent studies show anti-inflammatory and antioxidant activity of many marine sponge-derived sterols [12]. Therefore, the aim of the present study is to investigate the possible anti-inflammatory, antioxidant and immunomodulator effects of the methanolic extract of the Red Sea sponge *Xestospongia testudinaria*, which is not studied before for drug discovery on murine model of paw edema.

## Materials and Methods

### Sponge material

The sponge was collected in May 2013 from Ghurab reef (North side) in in the Red Sea at Jazan, Saudi Arabia at depths of 13–25 m, after permission from Saudi General Directorate of Border Guard. The collection of marine sponge *Xestospongia testudinaria* was conducted as a part of the research work supported by the King Abdulaziz University. We confirm that the filed studies included in this paper did not involve any endangered or protected species.

The sponge *Xestospongia testudinaria* is volcano-shaped with sharp lengthwise ridges on the pink or pale red-brown outer side. The surface layer of sponge skeleton is a tangential irregular isotropic or alveolar reticulation of spicule bundles of 2–6 spicules in cross section of about 20–60 μm thick and individual loose spicules. The bundles contain some binding sponging, which is barely visible. Thin growth stages of the spicules are oxeas. The spicule length is quite variable and measuring about 100–400 × 7–10 μm. The sponge was identified by Prof. Rob van Soest at the Naturalis Biodiversity Center at Leiden, The Netherlands. A fragment is kept in the collections of the Naturalis Biodiversity Center in Leiden, The Netherlands, under registration number RMNH Por. 9176. Another voucher specimen was deposited in our Red Sea Marine Invertebrates Collection, Faculty of Pharmacy, King Abdulaziz University under the code No. DY-KSA-12.

### Extraction and isolation

The freeze-dried sponge (180 g) was crushed and extracted by methanol (3 × 3 L). The crude extract was concentrated by evaporation under vacuum.

### Derivatization

About 1 mg of sample was mixed with 100 μL of CH_2_Cl_2_, vortexed, and dried with nitrogen gas. The residue was mixed with 50 μL N-methyl-N-trimethylsilyl-trifluoroacetamide (MSTFA), heated at 80°C for 15 min, cooled and a volume of 1 μL was injected for gas chromatography–mass spectrometry (GC–MS) analysis.

### Gas chromatography–mass spectrometry (GC–MS) analyses of the methanolic extract of *Xestospongia testudinaria*

The methanolic extract of *Xestospongia testudinaria* was analyzed by GC–MS. A Perkin Elmer Clarus 500 (Perkin Elmer, Shelton, CT, USA) was utilized throughout the experiments. The software controller/Integrator was TurboMass version 5.4.2.1617. An Elite-1 GC capillary column, Crossbond R at 100% dimethyl polysiloxane (30m×0.25mmid×0.25_mdf, Perkin Elmer) was used. The carrier gas was helium (purity 99.9999%) and the flow rate was 0.9 ml/min. Source (EI+): source temperature, 250°C. The GC line temperature was 200°C. Electron energy was 70 eV, and trap emission was 100 V. The oven was programmed as follows: initial temperature was 80°C (hold 5 min) to 250°C (rate 15°C/min, hold 5.0 min), followed by an increase to 280°C (rate 20°C/min, hold 2 min). The injector temperature was 260°C. The MS scan was from 45 to 350 *m/z* (500 scan/s). The injection volume was 1.0 μL, and the split ratio was 50:1. Samples were acquired by applying the positive total ion chromatogram (TIC). NIST2008 Program was used for matching characterized compounds.

### *In vitro* antioxidant assay (DPPH test)

The DPPH method was commonly applied to measure the antioxidant properties of natural compounds [13]. Different concentrations (125,250,500,1000 and 2000 μg/ml) of the *Xestospongia testudinaria* extract in methanol were prepared. 3 mL of DPPH (2,2-diphenyl-1-picryl-hydrazyl-hydrate) solution was mixed to 2 mL of each concentration. The mixtures were allowed to stand for 10-minutes at room temperature [14]. The absorbance was measured at 517 nm using a Thermo Scientific GENESYS 10S UV-visible double beam spectrophotometer (USA). Methanol was used as a blank and Tocopherol (vitamin E) was used as the positive control [15]. Lower absorbance of the reaction mixture indicate higher free radical scavenging activity. The following equation [15] shows the radical scavenging activity of the sample, which is stated as the inhibition percentage: % inhibition = [(Ac −A sample)/Ac] × 100, where Ac is the absorbance of the control (DPPH in absence of extract) and A sample is the absorbance in the presence of the extract.

### Animals and experimental design

Male Sprague Dawley rats (150–180 g) were used in this experiment. Animals were allowed free access to commercial pellet chow and water ad libitum. They were maintained at a temperature controlled room (22 ± 1°C) and humidity (55 ± 10%) with a 12 h light/dark cycle.

To minimize the suffering of laboratory animals, procedures including cervical dislocation were done under ether anaesthesia.

Thirty six rats were separated into six groups (n = 6). *Xestospongia testudinaria* methanolic extract (25, 50, and 100 mg/kg), indomethacin (Sigma, USA), the reference anti-inflammatory drug (10 mg/kg) [16], 0.9% saline, were injected intraperitoneal (i.p.) 2 h before carrageenan (Sigma, USA) injection. A noncarrageenan injected group was served as a control group. Animal study was implemented following the animal care and use Guideline for National Institutes of Health (NIH). The experimental protocol was approved by Unit of Biomedical Ethics Research Committee, Faculty of Medicine, King Abdulaziz University (Permit No:74–15).

#### Induction of paw edema in rat using carrageenan

The carrageenan-induced rat paw edema was performed according to the previously described technique [17]. Briefly, carrageenan (1.5% w/v, 0.1 ml/paw) was injected into right hind paw at the plantar side. Rats were observed for abnormal behavior and physical condition after carrageenan injection. The weight of the left (non-carrageenan injected) and right (carrageenan injected) paw was measured at 1, 2, 3, and 4 h of carrageenan injection [18]. For this purpose, rats were held firmly and each hind paw immersed in a beaker containing warm water (37°C) placed on a top-pan balance (Shinko Denshi Co.,Ltd.Japan) [18]. No deaths were reported throughout the experiment. Difference between the left and the right paw weight was obtained for assessment of edema at all the time points. The percentage increase in paw weight was calculated by dividing edema, weight after 1^st^, 2^nd^, 3^rd^ and 4^th^ hours by weight of left paw multiplied by 100.

#### Sample collection

Four h after carrageenan injection, 5 ml cardiac blood sample was taken into heparin from each rat under deep anesthesia, then the animal was killed by cervical dislocation. Hind paw specimens (left and right) were collected and Frozen at (-80°C) for estimation of lipid peroxides (measured as malondialdehyde, MDA), nitric oxide (NO), reduced glutathione (GSH) contents, glutathione peroxidase (GPx), superoxide dismutase (SOD) and catalase (CAT). After 3500 rpm centrifugation plasma aliquots were frozen at– 20°C for the analysis of TNFα, IL-1β and IL-6. Formalin fixed hind paw was used for the histopathologic examination.

### Measurement of paw lipid peroxide (measured as malondialdehyde (MDA)

MDA was measured using Biodiagnostic kits, Egypt, according to the method of Uchiyama and Mihara [19]. The color of the adduct formed following the reaction of thiobarbituric acid with the tissue homogenate in a boiling waterbath was measured at 535 nm. Paw MDA concentration was expressed as nmol/g tissue.

### Measurement of paw nitric oxide (NO)

The paw nitric oxide (NO) was measured using Biodiagnostic kits, Egypt, according to Tarpey et al. [20]. NO was determined in the liver homogenates using kits provided by Biodiagnostic, Egypt. Initially, nitrate was converted into nitrite by the enzyme nitrate reductase, followed by quantitation of nitrite using Griess reagent at the absorbance of 550 nm, as previously described [20]. NO was assayed by measuring total nitrate plus nitrite (NO3− +NO2−), the stable end products of NO metabolism. Paw NO concentration was expressed as μmol/g tissue.

### Measurement of paw reduced glutathione (GSH)

GSH was quantified using Biodiagnostic kits, Egypt, according to the method of Ellman [21]. The principle of this procedure is based on the formation of 2-nitro-5- mercaptobenzoic acid from reduction of bis(3-carboxy-4- nitrophenyl) disulfide reagent by the SH group, which has a deep yellow color that was measured spectrophotometrically at 412 nm. Paw GSH concentration was expressed as U/g tissue.

### Measurement of paw glutathione peroxidase enzyme activity (GPx)

Paw GPx activity was measured using Biodiagnostic kits, Egypt. GPx activity was determined in a coupled assay with glutathione reductase by measuring the rate of NADPH oxidation at 340 nm using H2O2 as the substrate [22]. GPx activity was expressed in U/g tissue.

### Measurement of paw superoxide dismutase enzyme activity (SOD)

Paw SOD activity was measured using Biodiagnostic kits, Egypt, according to the method of Nishikimi et al. [23]. This assay depends on the ability of the SOD to inhibit the phenazine methosulphate-mediated reduction of nitroblue tetrazolium dye. SOD activity was expressed in U/mg tissue.

### Measurement of paw catalase enzyme activity (CAT)

Paw CAT activity was measured using Biodiagnostic kits, Egypt, according to the method of Aebi [24]. H2O2 reacts with a known quantity of CAT. After adding catalase inhibitor, the reaction was stopped after exactly one minute. The remaining H2O2 reacts with 3,5-dichloro-2-hydroxybenzene sulfonic acid (DHBS) and 4- aminophenazone (AAP) to form a chromophore with color intensity inversely proportional to the amount of CAT in the original sample. The absorbance of samples was read at 510nm against a standard blank. CAT activity was expressed in U/g tissue.

### Measurement of plasma TNFα, IL-1β and IL-6

TNFα, IL-1β and IL-6 levels were measured in an ELISA assay as part of an Assaypro TNFα, IL-1β and IL-6 kits (30 Triad South Drive St. Charles, MO 63304, USA) using monoclonal antibodies specific for TNFα, IL-1β and IL-6, respectively. Cytokine concentrations were calculated using a standard purified recombinant cytokines.

### Histopathologic examination of the hind paws

Serial sections were taken from paraffin embedded paws and stained with hematoxylin and eosin (H&E), for the evaluation of histopathologic changes.

### Statistical analysis

One-way analysis of variance test (ANOVA) was used for comparison between different groups followed by Dunnett t-test (two-sided) multiple comparisons to detect significant differences among individual mean values of all groups. Results are expressed as the mean ± SDM. The level of significance was set at P ≤ 0.05. Statistical analysis was generated using SPSS software for windows, version 14.0 SPSS Inc., Chicago.

## Results

### Chemical constituents of methanolic extracts of *Xestospongia testudinaria*

**Fig 1:**
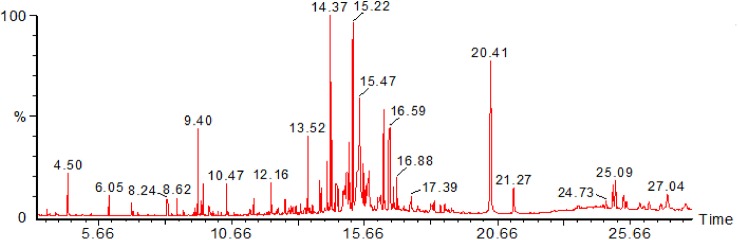
Chromatogram obtained from GC-MS with the methanolic extracts of the *Xestospongia testudinaria*.

Several organic compounds were detected and indicated. They include 3α-Trimethylsiloxy) cholest-5-ene (2.46), l-alanine (1.13), propanoic acid (1.92), pyrimidine (0.65), glutamine (0.14), azelaic acid (0.34), hexadecanoic acid (12.75), Tetradecanoic acid (2.11), Oleic acid (1.91) (4.77), Octadecadiynoic acid (3.25) and Octanoic acid (2.11) (Table 1 and Fig 1).

**Table 1:** Chemical constituents of *Xestospongia testudinaria* methanolic extract, detected by GC-MS.

Serial Number	Retention Time etention	Name of Compound	Peak Area (%)
1	3.79	Silane, trimethyl(3-phenoxypropoxy)-	0.64
2	4.03	N-Dimethylaminomethyl-tert.-butyl-isopropylphosphine	0.42
3	4.78	3-Heptene, 2,2,4,6,6-pentamethyl-	0.06
4	5.88	I-Trimethylsiloxy-2-trimethylsilylaminoethane	0.14
5	6.05	Propanoic acid, 2-[(trimethylsilyl)oxy]-, trimethylsilyl ester	1.92
6	6.89	l-Alanine, N-(trimethylsilyl)-, trimethylsilyl ester	1.13
7	7.12	Glycine, N-(trimethylsilyl)-, trimethylsilyl ester	0.35
8	8.62	L-Valine, N-(trimethylsilyl)-, trimethylsilyl ester	1.38
9	8.85	Butanoic acid, 4-[bis(trimethylsilyl)amino]-, trimethylsilyl ester	0.47
10	9.22	Silanol, trimethyl-, phosphate (3:1)	0.18
12	9.29	N,O-Bis-(trimethylsilyl)leucine	0.69
13	9.4	Trimethylsilyl ether of glycerol	6.22
14	9.49	Pentenoic acid, 4-[(trimethylsilyl)oxy]-, trimethylsilyl ester	0.89
15	9.54	L-Isoleucine, N-(trimethylsilyl)-, trimethylsilyl ester	1.93
16	9.82	Pyrimidine, 2,4-bis[(trimethylsilyl)oxy]-	0.65
17	9.97	2-Butenedioic acid (Z)-, bis(trimethylsilyl) ester	0.06
18	10.24	L-Serine, N,O-bis(trimethylsilyl)-, trimethylsilyl ester	0.24
19	10.47	Pentanedioic acid, bis(trimethylsilyl) ester	2.26
20	10.53	N,O,O-Tris(trimethylsilyl)-L-threonine	0.22
21	10.75	Octanoic acid, 7-oxo-, trimethylsilyl ester	0.32
22	11.37	Hexanedioic acid, bis(trimethylsilyl) ester	0.52
23	11.4	Pentonic acid, 2-deoxy-3,5-bis-O-(trimethylsilyl)-, γ-lactone	1.32
24	11.63	L-Aspartic acid, N-(trimethylsilyl)-, bis(trimethylsilyl) ester	0.12
25	11.99	L-Proline, 5-oxo-1-(trimethylsilyl)-, trimethylsilyl ester	0.24
26	12.16	Heptanedioic acid, bis(trimethylsilyl) ester	2.22
27	12.39	Glutamine, tris(trimethylsilyl)-	0.14
28	12.7	(±)-2-Hydroxyoctanoic acid, trimethylsilyl ester	1.08
29	12.98	D-Xylose, tetrakis(trimethylsilyl)-	0.51
30	13.26	Lyxose, tetra-(trimethylsilyl)-ether	0.88
31	13.57	Azelaic acid, bis(trimethylsilyl) ester	0.34
32	13.96	Tetradecanoic acid, trimethylsilyl ester	2.11
33	14.03	Oleic acid, trimethylsilyl ester	1.91
34	14.37	n-Pentadecanoic acid, trimethylsilyl ester	12.14
35	14.43	Undecanoic acid, 11-fluoro-, trimethylsilyl ester	3.48
36	15.07	Oleic acid, trimethylsilyl ester	4.77
37	15.22	Hexadecanoic acid, trimethylsilyl ester	12.75
38	18.15	1-O-hexadecylglycerol—bis-trimethylsilyl ether derivative	0.83
39	20.41	5,8,11-Eicosatriynoic acid, trimethylsilyl ester	18.78
40	21.27	9,12-Octadecadiynoic acid, trimethylsilyl ester	3.25
41	24.99	3α-(Trimethylsiloxy)cholest-5-ene	2.46

### Effect of *Xestospongia testudinaria* sponge extract on *In Vitro* Antioxidant Activity (DPPH Test)

**Fig 2:**
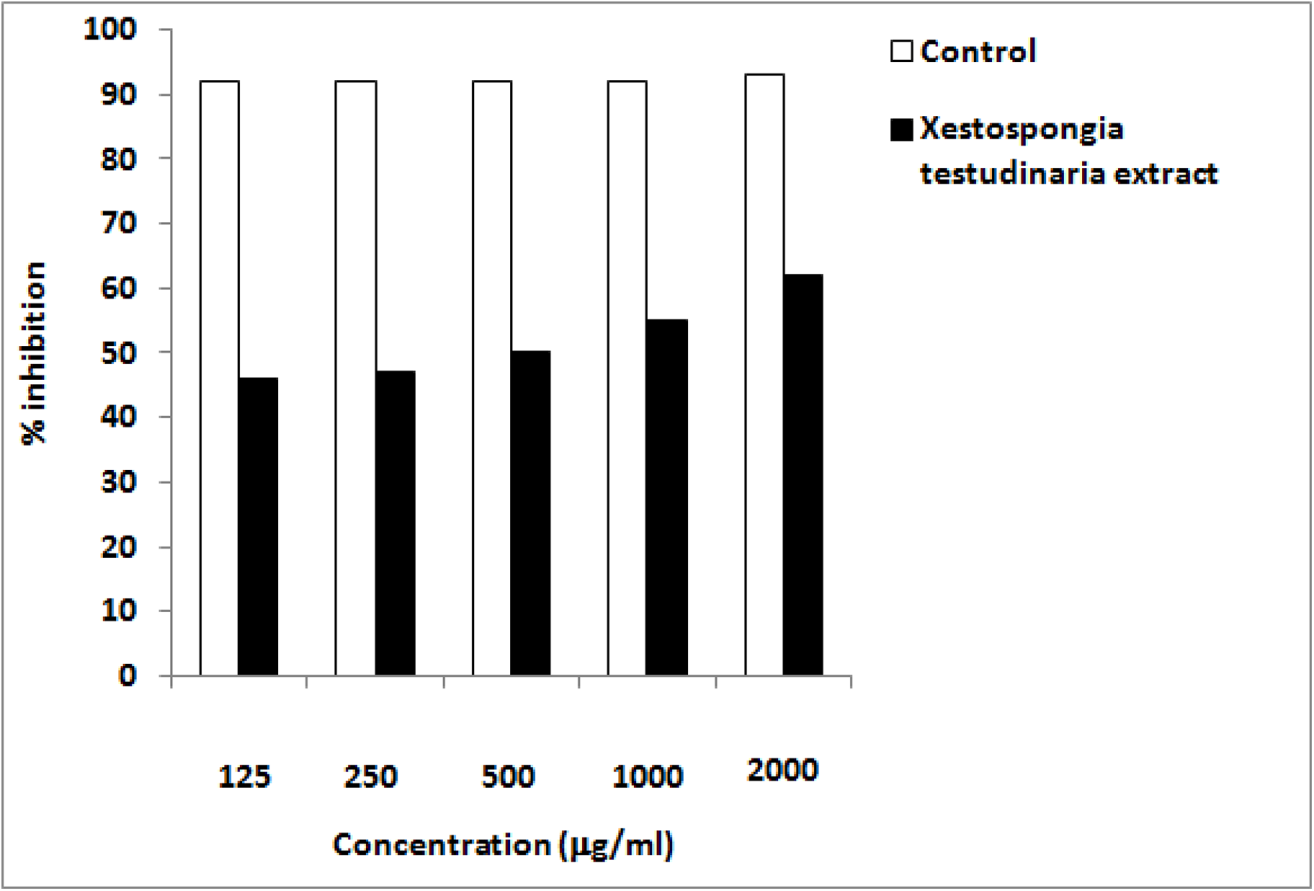
Total antioxidant capacity of *Xestospongia testudinaria* methanolic extract at different concentrations in vitro. Expressed as percent inhibition toward DPPH-induced oxidative stress.

The scavenging effects of the Red sea sponge methanolic extract, *Xestospongia testudinaria* on DPPH radicals increased with increasing concentration. The results were stated in terms of percentage of inhibition (Fig 2). The maximum percentage of inhibition was 62% at concentration of 2 mg/ml.

### Effect of methanolic extract of *Xestospongia testudinaria* (25, 50, and 100 mg/kg) and indomethacin (10 mg/kg) on carrageenan-induced rat hind paw edema (Fig 3)

Injection of rats with carrageenan caused a significant increase in the paw weight % measured after 1, 2, 3 and 4 h compared to the control values (p = 0.000). Pretreatment of carrageenan injected rats with indomethacin (10 mg/kg) significantly decreased paw weight % measured at 2, 3 and 4 h (29%, 46% and 31%, respectively) (p = 0.014, 0.009 and 0.019 respectively) compared to carrageenan injected rats. Pretreatment of carrageenan injected rats with *Xestospongia testudinaria* (25 and 50 mg/kg) did not decrease paw weight % measured at 1, 2, 3 and 4 h compared to carrageenan injected rats. On the other hand, pretreatment of carrageenan injected rats with *Xestospongia testudinaria* (100 mg/kg) significantly decreased paw weight % measured at 1, 2, 3 and 4 h (75%, 41%, 52% and 71% respectively) (p = 0.001, 0.000, 0.003 and 0.000 respectively) compared to carrageenan injected rats. In addition, *Xestospongia testudinaria* (100 mg/kg) significantly decreased paw weight % measured after 1 and 4 h of carrageenan injection (73% and 57%, respectively) (p = 0.002 and 0.004 respectively) compared to indomethacin injected rats.

**Fig 3:**
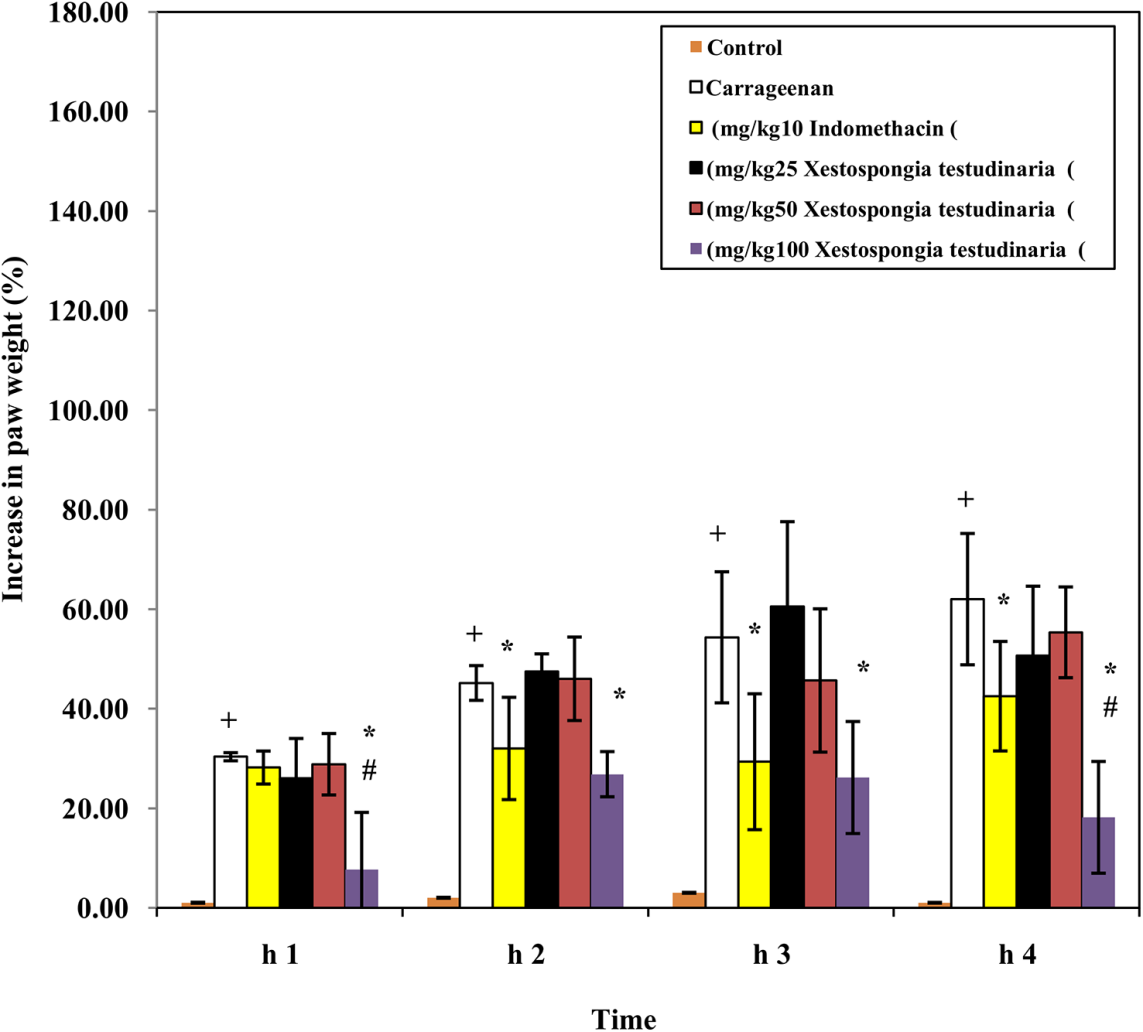
Effect of *Xestospongia testudinaria* methanolic extract (25, 50, and 100 mg/kg) and indomethacin (10 mg/kg) on carrageenan-induced rat hind paw edema. Results were expressed as a percentage increase in paw weight (mean ± SDM) of six rats. * Significant difference compared to carrageenan group (P ≤ 0.05). # Significant difference compared to indomethacin group (P ≤ 0.05).

### Effect of methanolic extract of *Xestospongia testudinaria* (100 mg/kg) and indomethacin (10 mg/kg) on paw lipid peroxides (MDA), nitric oxide (NO) and reduced glutathione (GSH) concentrations measured in carrageenan-induced rat hind paw edema

**Fig 4:**
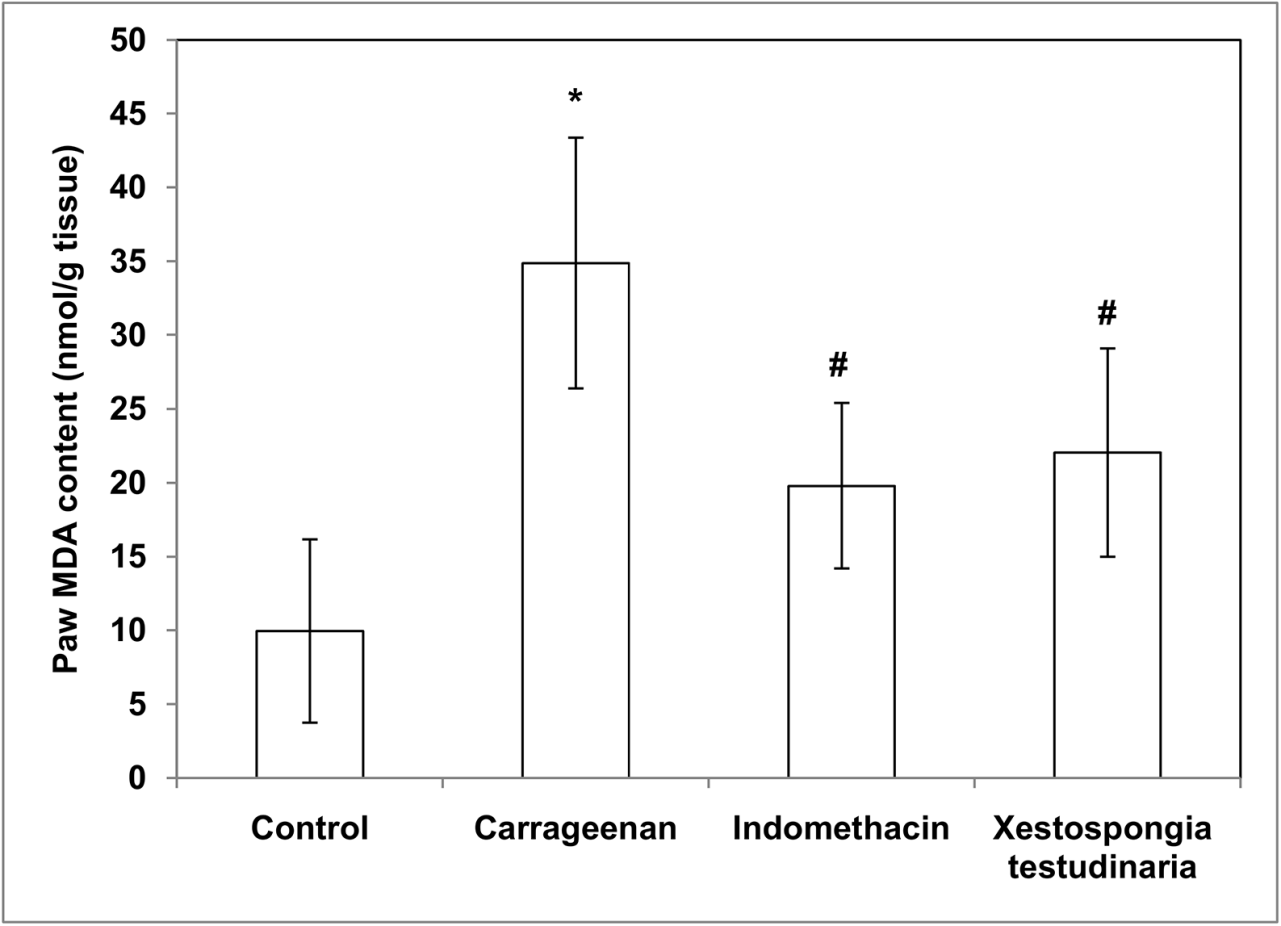
Effect of *Xestospongia testudinaria* methanolic extract (100 mg/kg) and indomethacin (10 mg/kg) on paw lipid peroxides (MDA) content (nmol/g tissue) measured in carrageenan-induced rat hind paw edema. Results were expressedas mean ± SDM of six rats.*Significant versus control (P ≤ 0.05). #Significant versus carrageenan (P ≤ 0.05).

**Fig 5:**
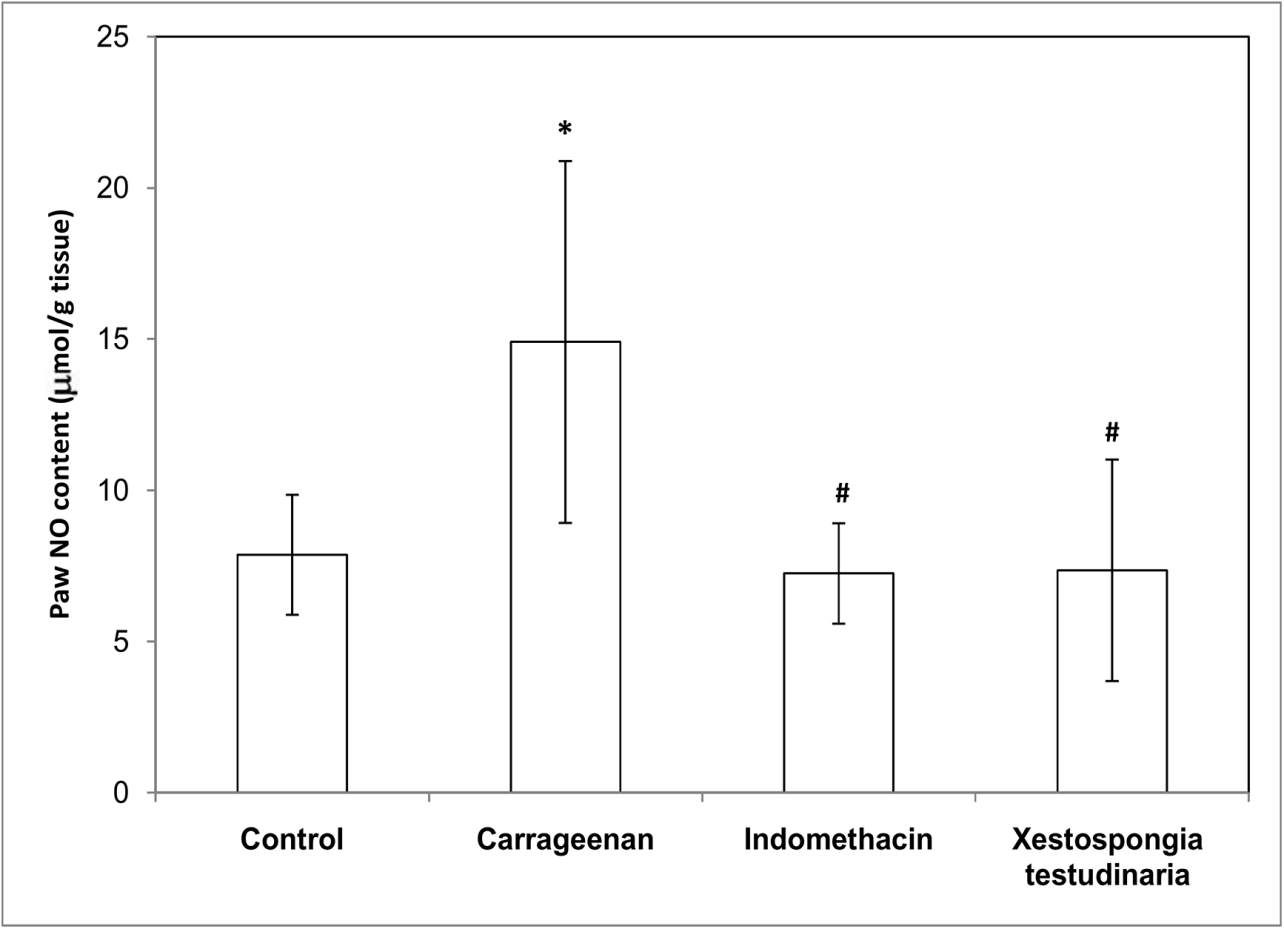
Effect of *Xestospongia testudinaria* methanolic extract (100 mg/kg) and indomethacin (10 mg/kg) on paw NO content (μmol/g tissue) measured in carrageenan-induced rat hind paw edema. Results were expressed as mean ± SDM of six rats. *Significant versus control (P ≤ 0.05).#Significant versus carrageenan (P ≤ 0.05).

**Fig 6:**
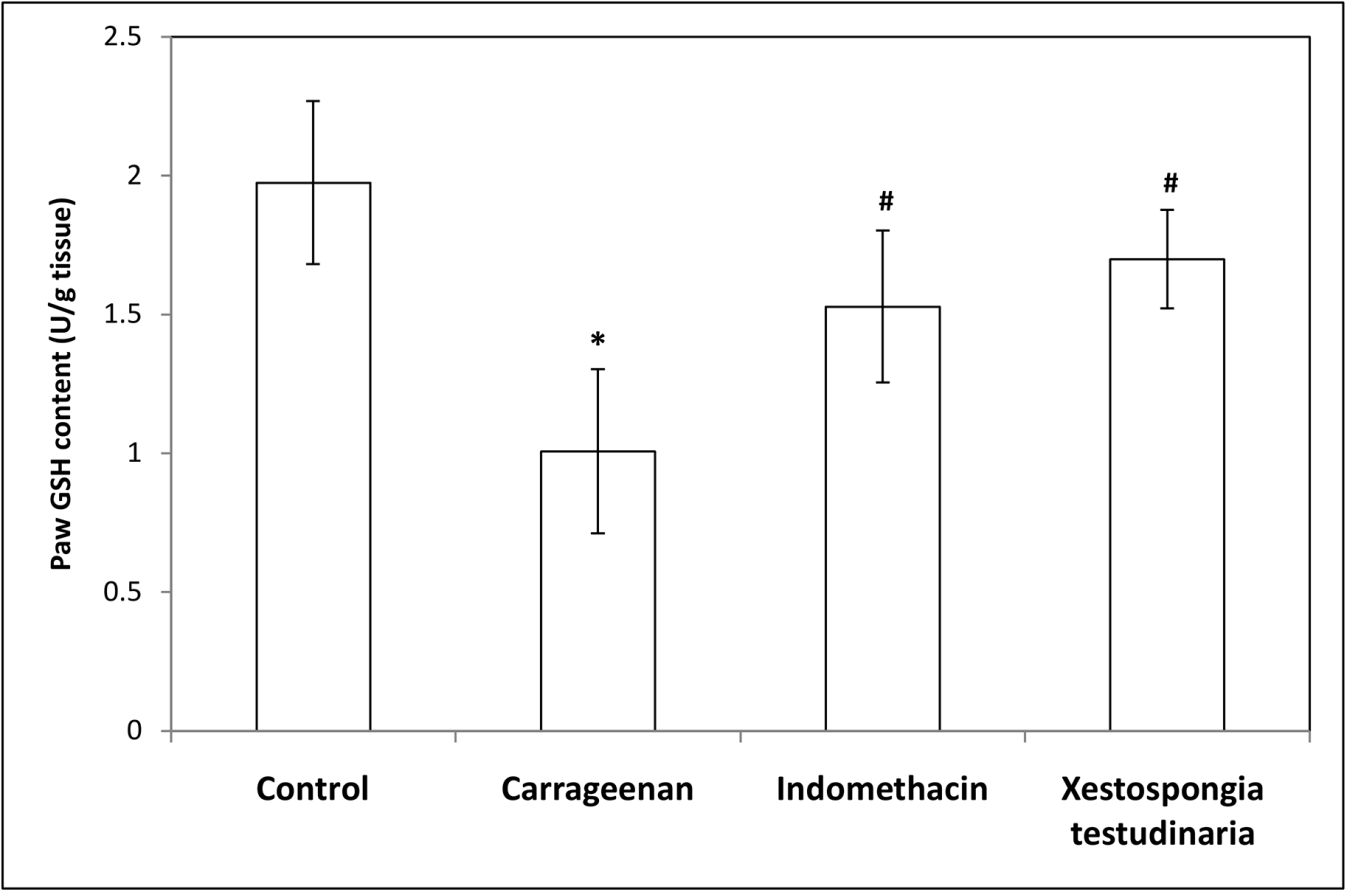
Effect of *Xestospongia testudinaria* methanolic extract (100 mg/kg) and indomethacin (10 mg/kg) on paw GSH content (U/g tissue) measured in carrageenan-induced rat hind paw edema. Results were expressed as mean ± SDM of six rats. *Significant versus control (P ≤ 0.05). #Significant versus carrageenan (P ≤ 0.05).

Treatments of rats with carrageenan caused a significant increase in both paw MDA and NO contents (~ 3.5 folds and 2 folds, respectively) compared to the control contents (p = 0.000 and 0.021, respectively) (Figs 4 and 5). On the other hand, treatments of rats with carrageenan caused a significant decrease (49%) in paw GSH contents compared to the control rats (p = 0.000) (Fig 6). Pretreatment of carrageenan injected rats with both *Xestospongia testudinaria* (100 mg/kg) and indomethacin (10 mg/kg) significantly decreased both paw MDA (37% and 43%, respectively) (p = 0.000) and NO contents (51% both) (p = 0.025 and 0.013 respectively) (Figs 4 and 5). On the other hand, pretreatment of carrageenan injected rats with both *Xestospongia testudinaria* (100 mg/kg) and indomethacin (10 mg/kg) significantly increased paw GSH contents (69% and 52%, respectively) (p = 0.001 and 0.010, respectively) compared to the carrageenan treated rats (Fig 6).

### Effect of methanolic extract of *Xestospongia testudinaria* (100 mg/kg) and indomethacin (10 mg/kg) on paw glutathione peroxidase (GPx), superoxide dismutase (SOD) and catalase (CAT) activities measured in carrageenan-induced rat hind paw edema

Briefly, the activities of SOD were not changed in all treatment regimens. Treatments of rats with carrageenan caused a significant decrease in both paw GPx (43%) and CAT activities (63%) compared to the control rats (p = 0.017 and 0.025, respectively). Pretreatment of carrageenan injected rats with both *Xestospongia testudinaria* (100 mg/kg) and indomethacin (10 mg/kg) significantly increased paw GPx (55% and 62%, respectively) (p = 0.021 and 0.046, respectively) and SOD enzymes activity (129% and 106%, respectively) (p = 0.000) compared to the carrageenan treated rats (Table 2).

**Table 2:** Effect of *Xestospongia testudinaria* methanolic extract (100 mg/kg) and indomethacin (10 mg/kg) on paw GPX, SOD and CAT enzymes activity measured in carrageenan-induced rat hind paw edema.

Treatment regimen	GPX(U/g tissue)	SOD(U/mg tissue)	CAT(U/g tissue)
**Control**	302 ± 94	0.22 ± 0.01	0.51 ± 0.29
**Carrageenan**	172 ± 58 ^a^	0.13 ± 0.01	0.19 ± 0.03 ^a^
**Indomethacin**	279 ± 91 ^b^	0.14 ± 0.04	0.39 ± 0.09 ^b^
***Xestospongia testudinaria* extract**	265 ± 61 ^b^	0.13 ± 0.04	0.43 ± 0.08 ^b^

Data are mean ± SD (n = 6).

^a^ Significant versus control (P ≤ 0.05).

^b^ Significant versus carrageenan (P ≤ 0.05).

### Effect of methanolic extract of the sponge *Xestospongia testudinaria* (100 mg/kg) and indomethacin (10 mg/kg) on plasma TNF-α, IL-1β and IL-6 measured in carrageenan-induced rat hind paw edema

Treatments of rats with carrageenan caused a significant increase in plasma TNF-α, IL-1β and IL-6 (30%, 12 folds and 106%, respectively) (p = 0.000) compared to the control rats. Pretreatment of carrageenan injected rats with both *Xestospongia testudinaria* (100 mg/kg) and indomethacin (10 mg/kg) caused a significant decrease in plasma TNF-α (18% and 17%, respectively) (p = 0.000 and 0.003, respectively), IL-1β (30% and 34%, respectively) (p = 0.000) and IL-6 (53% and 51%, respectively) (p = 0.000) compared to the carrageenan treated rats (Table 3).

**Table 3:** Effect of *Xestospongia testudinaria* methanolic extract (100 mg/kg) and indomethacin (10 mg/kg) on plasma TNF-α, IL-1β and IL-6 measured in carrageenan-induced rat hind paw edema.

Treatment regimen	TNF-α(pg/ml)	IL-1β (pg/ml)	IL-6 (pg/ml)
**Control**	309 ± 20	127 ± 36	129 ± 7
**Carrageenan**	402 ± 26 ^a^	1529 ± 159 ^a^	266 ± 21 ^a^
**Indomethacin**	333 ± 33 ^b^	1011 ± 106 ^b^	131 ± 5 ^b^
***Xestospongia testudinaria* extract**	330 ± 17 ^b^	1076 ± 61 ^b^	124 ± 2 ^b^

Data are mean ± SD (n = 6).

^a ^Significant versus control (P ≤ 0.05).

^b^ Significant versus carrageenan (P ≤ 0.05).

### Histopathological study

**Fig 7:**
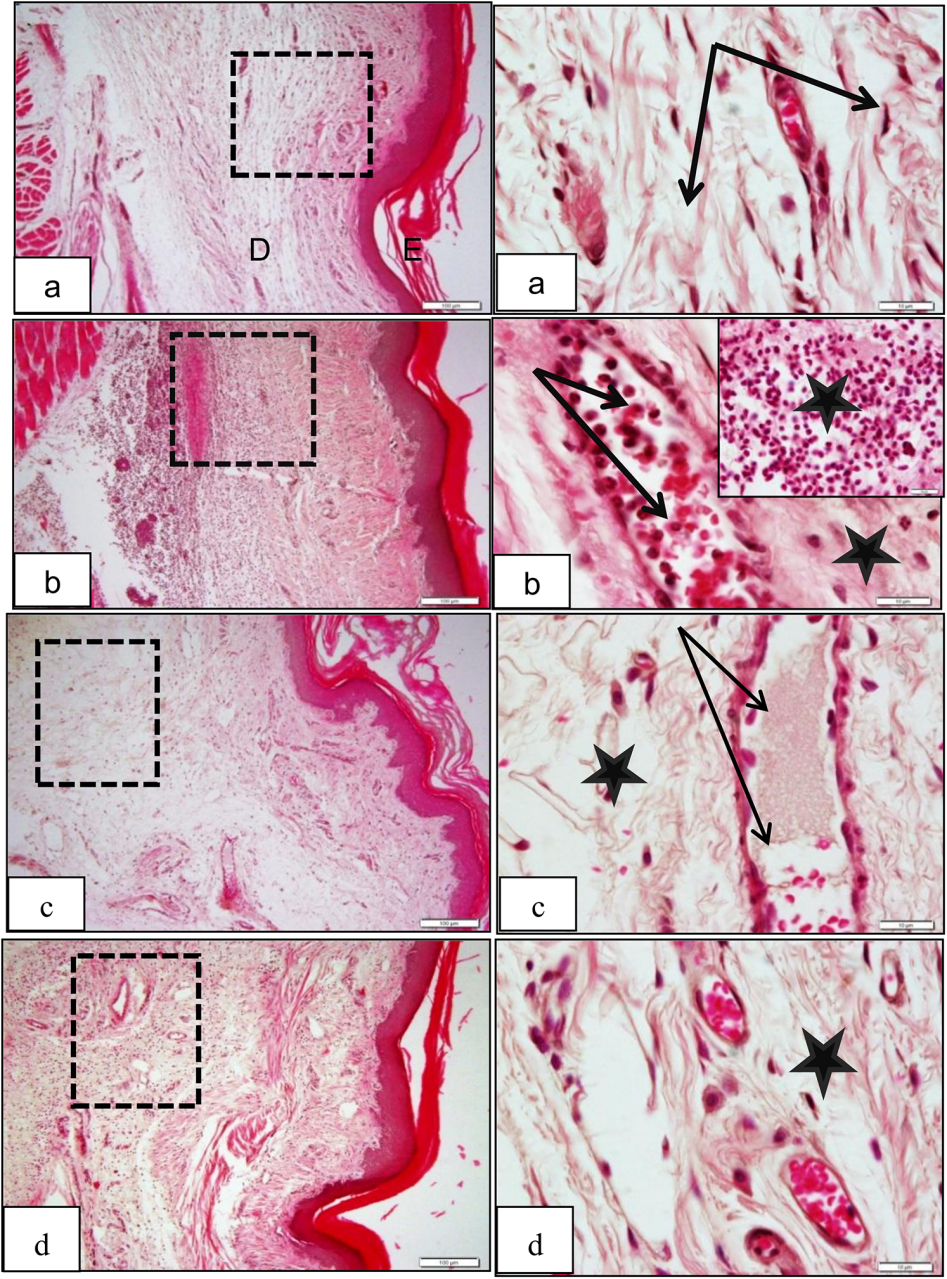
Low and magnified power of rat paw sections stained by H&E, (a) Control group, E: epidermis, D: dermis with no signs of vascular congestion or inflammatory cells (dotted square) with normal capillaries (black arrows) and connective tissue dermis (stars). (b) Carrageenan group, showing marked inflammatory infiltration in the deep dermis (D), capillary dilation and congestion with neutrophils margination prior to escape into the surrounding tissue (arrows;) (c) *Xestospongia testudinaria* methanolic extract group, showing significant decrease of the inflammatory cells in the dermis and within blood vessels. (d) Indomethacin group, showing a decrease in both inflammatory cells infiltration and vascular congestion.

Carrageenan injections into rat paw induced a good model of acute inflammatory reaction that characterized by blood capillary dilation and congestion with marked inflammatory exudate consists mainly of polymorphnuclear leucocytes and occasionally mast cells (Fig 7B). Injection of *Xestospongia testudinaria* (100 mg/kg) 2 h before carrageenan injection exerted marked anti-inflammatory effect represented by marked decrease in the capillary congestion and inflammatory cells infiltrate (Fig 7C) which is more or less similar to the action of the reference anti-inflammatory drug, indomethacin (10 mg/kg) (Fig 7D).

## Discussion

Assessment of anti-inflammatory activity of marine natural products from sponges using carrageenan-induced inflammation in the rat paw is the most common assay [25,26]. In the current study, *Xestospongia testudinaria* methanolic extract revealed significant anti-inflammatory activity at a dose of 100mg/ kg even than the indomethacin treated group. Several compounds possess varied chemical structures and potent anti-inflammatory activity has been isolated from marine sponges [9]. In the present study, GC-MS analysis revealed five compounds exhibit antiinflamatory activity, Propanoic acid [27], Pyrimidine [28], Glutamine [29]. Also GC-MS revealed the presence of several fatty acids as (Tetradecanoic acid, Oleic acid, Octadecadiynoic acid, Octanoic acid) with sterol derivative 3α-(Trimethylsiloxy) cholest-5-ene. Pervious study on sea star found that, maximum antiinflammatory activity was obtained after combination of steroid and fatty acid [30]. Hexadecanoic acid possesses anti-inflammatory and antioxidant activity [31,32].

Recently, five sterols (cholesterol, 24-hydroperoxy-24-vinylcholesterol, saringosterol, methylcholest-5-ene-3β, 25-diol, and 29-hydroperoxystigmasta-5,24(28)-dien-3β-ol) were isolated from the marine sponge *Xestospongia testudinaria* [11]. Recently, we reported that the Red Sea sponges *Scalarispongia aqabaensis* and *Callyspongia siphonella* produced two new sterols, which exhibit anti-inflammatory activity using paw induced edema in rat [33]. *Xestospongia testudinaria* methanolic extract also had antioxidant activity as revealed by DPPH assay. GC-MS showed the presence of Azalic acid and l-Alanine which could be responsible for the antioxidant activity of *Xestospongia testudinaria* sponge extract [34,35]. ROS such as superoxide anion, hydroxyl radicals and hydrogen peroxide play major roles in terms of producing cellular damage in the inflammatory processes [36]. NO and MDA, the end product of lipid peroxidation [37], are considered important markers of oxidative stress [37–39]. In the present study, paw MDA and NO were increased while GSH, GPx, CAT and SOD were reduced in the carrageenan inflamed group. The results of the current work revealed that both *Xestospongia testudinaria* methanolic extract and indomethacin reduced paw MDA and NO and increased GSH formation. Moreover, both elevated the decreased activities of the antioxidant enzymes, GPx and CAT. GPx, in the existence of GSH are considered the second line defense against hydroperoxides where it accelerates hydrogen peroxide reduction or other organic hydroperoxides [40]. Phytochemicals present in sponges can act as antioxidants and prevent disorders due to oxidative damage [41]. In our recent published work, the extract of the Red Sea sponge *Suberea mollis* exhibited a significant antioxidant activity against CCl_4_-induced oxidative stress in rat liver [42]. Furthermore, sterols isolated from the sponge Fascaplysinopsis *sp*. are found to display antioxidant activity [43].

Inflammatory cytokines TNF-α, IL-1β and IL-6 play an important role in the inflammatory process and are responsible for production of acute phase proteins [44]. Treatment strategies are based on the presence of these inflammatory cytokines [45]. TNF-α, IL-1β and IL-6, are released mainly from monocytes and macrophages at the inflammatory sites [46]. In the present study, pretreatment of carrageenan-treated rats with *Xestospongia testudinaria* methanolic extract or indomethacin result in reduction in elevated TNF-α, IL-1β and IL-6. Previous studies found that proactive metabolites isolated from marine sponge-derived fungus *Penicillium* species, inhibit TNF-α, IL-1β and IL-6 in murine macrophage cells [47]. Similarly, marine sponge metabolite hymenialdisine was found to be a potent inhibitor of IL-2 and TNF-α production [48].

## Conclusion

The results of this study demonstrated that pretreatment with the methanolic extract of the Red Sea marine sponge *Xestospongia testudinaria* prevents carrageenan-induced acute local inflammation in rats. The protective effect is based on the augmentation of antioxidant enzyme system which in turn causes a reduction in the oxidative stress measures. *Xestospongia testudinaria* methanolic extract may also prevent acute local inflammation through inhibition of proinflammatory cytokines formation.

## Recommendation

Future study is warneted with orally consumed extract instead of injected intraperitoneal. In addition, bioassay guided fractionation of the crude *Xestospongia testudinaria* methanolic extract is important.
